# Primary carcinosarcoma of the liver: imaging features and clinical findings in six cases and a review of the literature

**DOI:** 10.1186/s40644-018-0141-0

**Published:** 2018-02-27

**Authors:** Jing Li, Pan Liang, Dandan Zhang, Jie Liu, Hongkai Zhang, Jinrong Qu, Jianbo Gao

**Affiliations:** 1 Department of Radiology, the Affiliated Cancer Hospital of Zhengzhou University, Henan Cancer Hospital, No. 127, Dongming Road, Zhengzhou, Henan 450008 China; 2 Department of Radiology, the First Affiliated Hospital of Zhengzhou University, No. 1, East Jianshe Road, Zhengzhou, Henan 450052 China; 3 Department of Pathology, the First Affiliated Hospital of Zhengzhou University, No. 1, East Jianshe Road, Zhengzhou, Henan 450052 China

**Keywords:** Liver, Carcinosarcoma, Tomography, X-ray computed, Magnetic resonance imaging, Diagnosis

## Abstract

**Background:**

Carcinosarcoma of the liver is a very rare tumor composed of a mixture of carcinomatous and sarcomatous elements. Less than 25 adequately documented cases have been reported, with inadequate description of imaging features. In order to improve the awareness of this rare tumor, this study aimed to analyze the clinicopathologic and imaging features of six cases of hepatic carcinosarcoma (HCS) confirmed by surgical pathologic evaluation.

**Methods:**

We retrospectively studied the clinicopathologic and imaging features of six cases of HCS (matching the World Health Organization definition) and discussed the differential diagnosis on the basis of imaging findings. The patients, including five men and one woman, were 38 to 69 years of age. Five patients underwent CT scans, one underwent MRI scans.

**Results:**

While 3 patients were positive for hepatitis-B surface antigen, 2 had cirrhosis. The largest tumor diameter ranged from 5.0 to 21.0 cm. Satellite nodules, venous thrombi, and organ invasion (gastric wall, gallbladder, and right adrenal gland) were identified. Pathologically, the carcinomatous components corresponded to hepatocellular carcinoma in three cases, cholangiocellular carcinoma in one case, and adenocarcinoma in two cases. The sarcomatous components exhibited complex features, with undifferentiated spindle cells in five cases and a leiomyosarcoma in one. All tumors showed heterogeneous density/intensity with extensive cystic change and necrosis; spot calcification was observed in one case. Capsule was not identified. While four tumors showed heterogeneous hypervascular enhancement, two showed hypovascular enhancement. All patients underwent surgical resection. The follow-up period ranged from 2 to 18 months. Four patients died from recurrence and metastasis.

**Conclusions:**

The clinical and imaging features of HCS are heterogeneous. Due to the heterogenous nature and very low morbidity of HCS, combination of careful analysis of imaging findings and clinical features might be useful for a more accurate diagnosis of HCS.

## Background

Primary hepatic carcinosarcoma (HCS) is a very rare malignant tumor composed of a mixture of carcinomatous and sarcomatous elements [1]. This tumor is difficult to diagnose clinically; it is also aggressive and has poor prognosis, being associated with a high frequency of early metastasis and advanced stage at diagnosis [2].

To date, only 23 cases of primary HCS have been reported in the English medical literature [2–18]. To the best of our knowledge, the majority of these studies were case reports, focused on the pathogenesis and pathological diagnosis and prognosis of HCS. Only 10 of these studies briefly described the imaging features of HCS [4, 6–9, 13, 14, 16–18], there is much unkown about this rare tumor. Due to its more aggressive nature and poorer prognosis than pure HCC and CCC, it is clinically beneficial to narrow down the differential diagnoses by accumulating imaging findings of HCS. This study analyzes the imaging findings and clinical features of six cases of HCS and also presents a review of the literature.

## Methods

### Patient selection

Between January 2013 and 2017, we searched the pathology records and the Picture Archiving and Communication Systems at our institution using the following keywords: “hepatic sarcomatoid carcinoma”, “hepatic sarcoma”, and “hepatic carcinosarcoma”. We identified 767 consecutive patients with pathologically proven liver primary malignancies, of whom only 6 patients had been diagnosed with liver carcinosarcoma by surgical pathologic evaluation in accordance with the World Health Organization (WHO) definition of 2000. Data of these 6 cases of carcinosarcoma were retrieved from institutional and consultation files. Clinical information was extracted from patient records, and follow-up data were obtained from the physicians. Pathology reports, clinical data (including demographic features, laboratory findings, clinical intervention, and treatment outcome), and imaging data were reviewed. This study was approved by the ethics committee at our institution.

### Image acquisition

#### CT protocol

Five patients underwent CT scans. In the CT study, unenhanced and dual-phase contrast-enhanced (CE) CT images were acquired with a 64-row multidetector CT scanner (Discovery CT 750 HD, GE Healthcare, Waukesha, Wisconsin, USA). The imaging study was performed from the diaphragm to the iliac crest. The imaging parameters were as follows: tube voltage, 120 kV; tube current, 350 mA; rotation time, 0.5 s; field of view (FOV), 48 mm; matrix, 512 mm; and section thickness, 0.75 mm. A total of 70–120 mL (1.5 mL/kg) of a nonionic contrast medium (Ultravist 370, Bayer Schering Pharma, Berlin, Germany) was injected at a rate of 3.0 mL/s through the antecubital vein using a dual-head pump injector (Medrad, Warrendale, Pennsylvania, USA). Finally, 20 mL saline flush was injected at a rate of 3 mL/s. Each dataset was reconstructed with 5 mm thickness. Contrast enhanced CT images were acquired with a scanning delay of 30 s (arterial phase, AP) and 70 s (portal venous phase, PP) after the start of intravenous (i.v.) contrast injection.

### MRI protocol

One patient underwent magnetic resonance (MR) scan. Upper abdomen MR unenhanced and dynamic enhanced images were acquired using a 3.0 T MR scanner (Signa HDx, GE Healthcare, Waukesha, Wisconsin, USA) with a torso coil. Patients were positioned supine, feet first. The main imaging sequences included the followings: (1) spoiled gradient-echo, breath hold, three-dimensional double-echo steady state (SPGR-BH-3DDE) axial T1-weighted imaging (T1WI) —in-phase: repetition time (TR), 3.98 ms; echo time (TE), 2.34 ms; opposed-phase: TR 3.98 ms, TE 1.17 ms; slice thickness, 4 mm; section gap, 0.4 mm; FOV, 38 × 38 cm; matrix, 260 × 180; and flip angle, 12°; (2) turbo spin echo (TSE) fat-suppression T2-weighted imaging (T2WI) — TR, 7500.0 ms; TE,118.4 ms; slice thickness, 7 mm; section gap, 0.7 mm; flip angle, 90°; FOV, 38 × 38 cm; and matrix, 224 × 288; and (3) liver acquisition with volume acceleration (LAVA) dynamic enhanced imaging — TR, 2.6 ms; TE, 1.4 ms; slice thickness, 4 mm; flip angle, 12°; FOV, 38 × 38 cm; and matrix, 170× 272. The contrast agent gadolinium-diethylenetriamine pentaacetic acid (Gd-DTPA; Magnevist, Bayer Schering Pharma, Berlin, Germany) was injected with a dosage of 0.1 mmol/kg into the antecubital vein through a pump injector (Medrad, Warrendale, Pennsylvania, USA) at a flow rate of 2.5 mL/s, after which 20 mL saline was injected at the same rate. Enhanced MRI series included six phases, including two continuous scans each at 12 s and 50 s and one scan each at 90 s and 150 s after contrast agent injection. One scanning phase lasted for 7–8 s.

### Image analysis

All image analyses were performed independently at an ADW4.5 workstation (GE Healthcare, Waukesha, Wisconsin, USA) by two radiologists (with 7 and 15 years of experience in abdominal radiology) blinded to the clinical information. The evaluated parameters included tumor site (segment or lobe), size (long-axis diameter), shape (bulky, round, or multilocular), margin (clear or unclear), density/intensity (hypo-, iso-, or hyperdense/intense relative to normal liver), lesion texture (homogeneous or heterogeneous), cystic/necrotic degeneration, calcification, hemorrhage, capsule (present or absent), and enhancement characteristics, including enhancement pattern (homogeneous or heterogeneous) and degree (hyper, or hypo-vascular). Cystic and necrotic areas were defined by water density/intensity without enhancement on CT/MR images. Enhancement degree of a tumor was determined by comparison of density/intensity between the tumor and hepatic parenchyma in CE images; tumors with density/intensity greater or lower than that of normal liver were adjudged to be “hypervascular” or “hypovascular”, respectively. Regions of interest were placed at the center of the mass, avoiding areas of cystic/necrotic changes, vessels, calcification, and hemorrhage. All measurements were repeated three times at the three contiguous imaging levels, and average values were calculated to ensure consistency. Data regarding vascular invasion, lymphadenopathy, bile duct involvement, invasion of adjacent organs, presence of fluids (ascites or pleural effusion), and pretreatment image diagnoses were also recorded. Patient age, gender, symptoms, disease duration, and laboratory findings were also reviewed. Finally, findings were compared with the pathological results.

### Pathological evaluation

Pathological images were reviewed by two pathologists independently. According to the WHO definition, HCS is “a malignant tumor containing an intimate mixture of carcinomatous(either hepatocellular or cholangiocellular) and sarcomatous elements”, and morphological findings from hematoxylin–eosin (HE)-stained sections should be considered in combination with both carcinomatous and sarcomatous markers [8]. Immunohistochemistry (IHC) analysis of paraffin-embedded sections was performed by the avidin–biotinylated peroxidase complex method. Antibodies used in this study included epithelial markers such as AE1/AE3, CK5/6/7, CK8, CK18/19, hepatocytes, and α-fetoprotein (AFP); mesenchymal markers included vimentin, myogenin, desmin, striated muscle actin (SMA), epithelial membrane antigen (EMA), and cluster of differentiation (CD) 117 (c-kit); and other markers, such as S100, CD31, CD34, and CD68. All antibodies listed above were purchased from DAKO (Dako, Glostrup, Denmark).

### Literature review

The PubMed and Medline databases were searched, with language restriction, for the following terms: (Hepatic [MeSH]) AND Carcinosarcoma [MeSH]) and (Liver [MeSH]) AND Carcinosarcoma [MeSH]).The search retrieved 17 full text articles with 23 cases of HCS. Of the 17 studies, 10 had briefly summarized the imaging features of HCS. The publication period ranged from Jan 1989 to Jan 2016.

## Results

### Clinical characteristics

The six selected patients included five men and one woman, with a median age of 54.5 years (age range, 38–69 years). While three patients (nos.1, 5, and 6) were positive for hepatitis-B virus (HBV) antibodies, two (nos.1 and 5) had cirrhosis at the time of administration. Laboratory findings revealed patients 1 and 4 to be positive for carbohydrate antigen 125(CA125) and 19–9(CA19–9), patient 4 to be positive for carcinoembryonic antigen (CEA), and patients 1 and 2 to be positive for AFP (Table 1).

**Table 1:** Preoperative clinical features of 6 cases with hepatic carcinosarcoma

Case NO.	Age(y)	Gender	Symptom and Duration	HBV	HCV	Cirrhosis	CA125	CA19–9	CEA	AFP
1	45	M	Fever and abdominal distension, 2 months	+	–	+	+	+	–	+
2	69	F	Fever and anorexia, 3 months	–	–	–	–	–	–	+
3	38	F	Right upper quadrant discomfort, 3 months	–	–	–	–	–	–	–
4	61	M	abdominal distension and weight loss, 3 months	–	–	–	+	+	+	–
5	48	M	Fever and abdominal distension, 2 months	+	–	+	–	–	–	–
6	68	M	Abdominal pain and distension	+	–	–	–	–	–	–

*Abbreviation*: *M* male, *F* female, *AFP* α-fetoprotein (positive defined as > 25 ng/mL), *HbsAg* Hepatitis B surface antigen, *HCV* Hepatitis C Virus, *CA125* carbohydrate antigen 125 (positive defined as > 35 U/mL), *CA19–9* carbohydrate antigen 19–9 (positive defined as > 35 U/mL), *CEA* carcinoembryomic antigen (positive defined as > 25 U/mL), “+” yes/present/positive, “-” no/absent/ negative

### Pathological features and follow-up data

All six patients underwent surgical resection, and the diagnoses were confirmed on the basis of surgical pathologic and IHC findings (Table 2). Three patients (nos.1, 4, and 6) underwent hemihepatectomy; patient 6 underwent additional right adrenal resection. Patient 2 underwent segmentectomy. In case of patient 3, segmentectomy, cholecystectomy, and partial gastrectomy were performed because of gallbladder and gastric wall invasion of the lesion. Patient 5 underwent lobectomy. All six patients recovered without complications after hepatectomy and were discharged from the hospital 12–18 days post-surgery. All patients were followed-up until March 26, 2017. Postoperative survival time ranged from 2 to 18 months. Recurrence free survival time ranged from 1.5 to 18 months. Four patients (nos.2, 3, 5, and 6) developed recurrence and metastasis (patient 2: right lung and mediastinal nodes; patient 3: liver and retroperitoneal nodes; patient 5: liver; and patient 6: liver, right lung lobe, and retroperitoneal lymph nodes); these four patients died during the follow-up period. Patients 1 and 4 showed no recurrence and were alive at the end of the follow-up period.

**Table 2:** Pathological features and follow-up data for 6 patients diagnosed with carcinosarcoma

Case NO.	Maximum diameter (cm)	Satellite Nodules	Vessel Thrombi	Extrohepatic lesions	Surgical procedure	Components	IHE positive marker for carcinomatous element	IHE positive marker for sarcomatous element	Recurrence free survival (months)	Recuurence/ metastasis?	Follow-up period (months)/outcome
1	21	–	–	–	Left hemihepatectomy	HCC + spindle cells	AE1/AE3, CK8/18, Hep, AFP	Vim	2	–	2/A
2	14.5	–	–	–	Segmentectomy	CCC+ spindle cells	CK7/8	Vim	7	+/Right lung, mediastinal lymph nodes	13/D
3	7.5	–	–	gastric wall, gallbladder	Segmentectomy, cholecystectomy,partial gastrectomy	HCC+ spindle cells	CK5/6/7	Vim	1.5	+/Liver, retroperitoneal lymph nodes	8/D
4	5	–	–	–	Left hemihepatectomy	Adenocarcinoma + leiomyosarcoma	CK7/18/19	Vim, SMA	18	–	18/A
5	7.5	–	–	–	Lobectomy	HCC+ spindle cells	Hep	Vim	8	+/Liver	18/D
6	10.5	one in S6	right hepatic vein	right adrenal	Right hemihepatectomy, right adrenal resection	Adenocarcinoma + spindle cells	CK18	Vim	2	+/Liver, right lung, and para-aortic lymph nodes	4/D

*Abbreviation*: “+” yes/present/positive, “-” no/absent/ negative, *CK* cytokeratin, *Hep* hepatocytes, *Vim* vimentin, *SMA* striated muscle actin, *S6* segment 6, *D* died, *A* alive

**Fig. 1:**
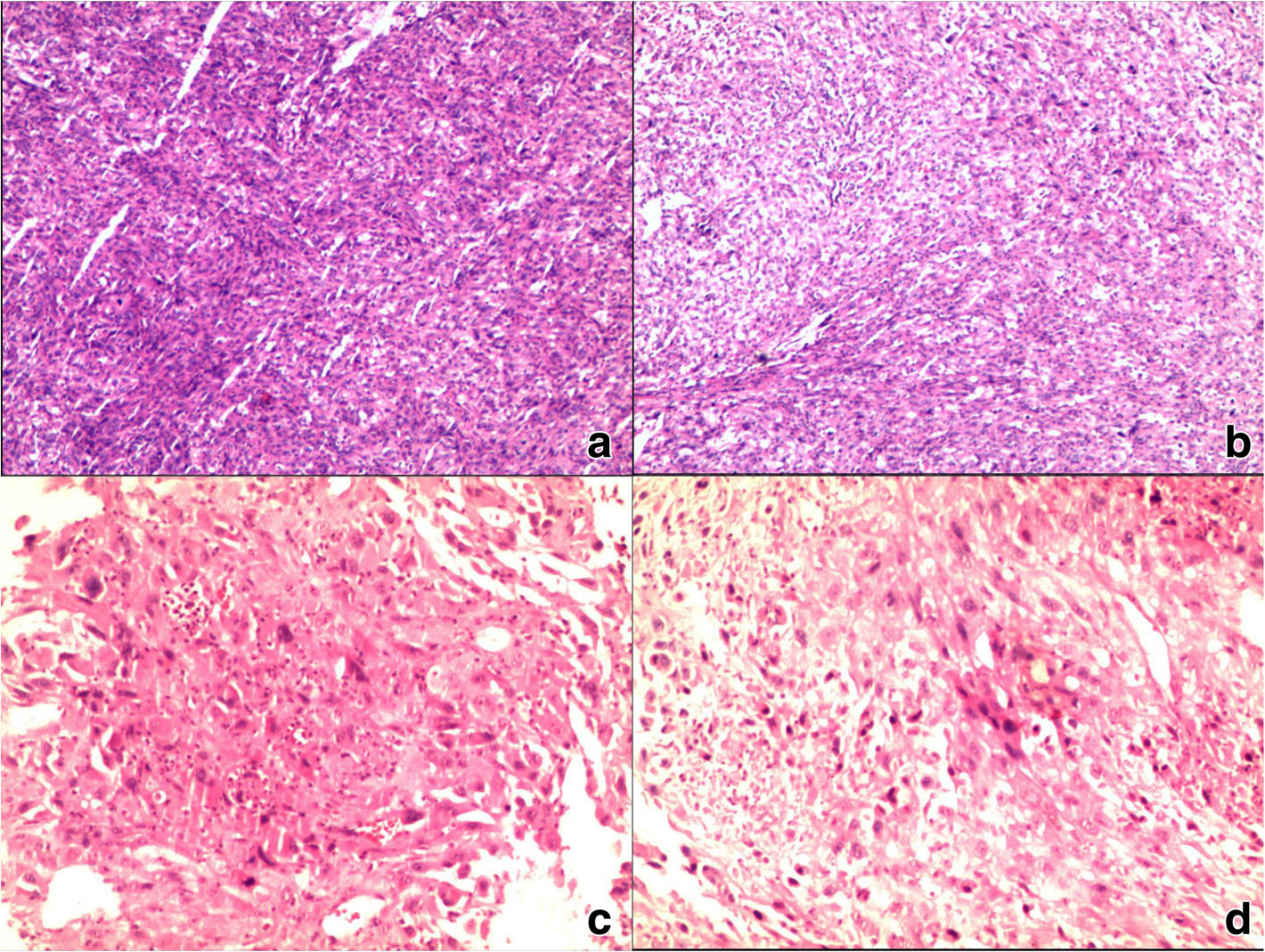
Histological appearance (hematoxylin and eosin stain) of HCS. **a** Histological appearance of No. 5 tumor shows poorly differentiated hepatocellular carcinoma in the carcinomatous component. **b** Sarcomatous component shows undifferentiated spindle cells. **c** Histological appearance of No. 4 tumor (hematoxylin and eosin stain) shows a not otherwise-specified adenocarcinoma in the carcinomatous component. **d** Sarcomatous component shows undifferentiated spindle cells

**Fig. 2:**
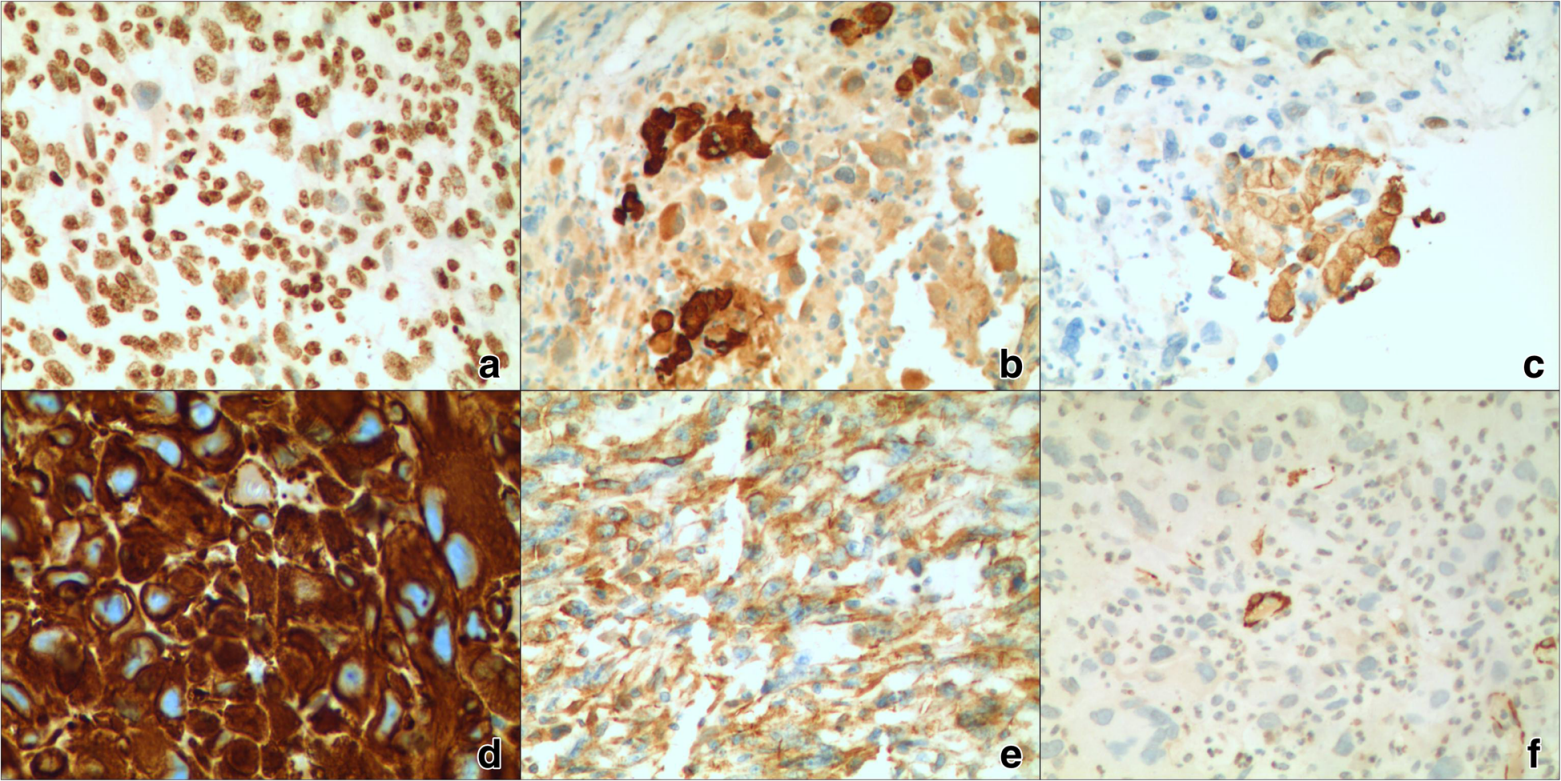
Immunohistochemical expressions in patient with HCS. Hepatocytes (**a**) and CK (**b**), CK18(**c**) is positive in the hepatocellular carcinomatous component. Vimentin (**d, e**) and SMA (triated muscle actin) (**f**) are positive in the sarcomatous component

In all six cases, the largest tumor dimensions were no less than 5.0 cm. Sectioned surfaces were heterogeneous because of secondary changes, including hemorrhage and necrosis. Microscopically, all tumors contain identifiable malignant epithelial (carcinomatous) and mesenchymal (sarcomatous) components with moderate to poor differentiation. Most of the neoplastic cells were spindle-shaped or pleomorphic, arranged in variable architectures such as fascicles, sheets, whorls, and storiform patterns (Fig. 1). Sarcomatous cells were spindle-shaped, with poorly differentiated or anaplastic features (Fig. 1). The carcinomatous components corresponded to hepatocellular carcinoma (HCC) in three cases, cholangiocellular carcinoma (CCC) in case of patient 2, and a “not otherwise specified” adenocarcinoma in case of patient 4 and 6. IHC results showed positive expression in AE1/AE3, hepatocyte, AFP, and CK5/6/7/8/18/19 antibodies for epithelial component (Table 2) (Fig. 2a, b, c). Vimentin, a common sarcomatous marker (Fig. 2d, e), was positive in all tumors. In case of patient 4, the tumor exhibited specialized differentiation substantiated by SMA positive expression and was diagnosed as a leiomyosarcoma (Fig. 2f). According to the surgical results, patients 3 and 6 exhibited extrahepatic lesions and tumor invasion of the gastric wall, gallbladder, and right adrenal gland, respectively. Patient 6 also exhibited vessel thrombi in the right hepatic vein and an intrahepatic satellite node in segment 6.

### Imaging findings

**Fig. 3:**
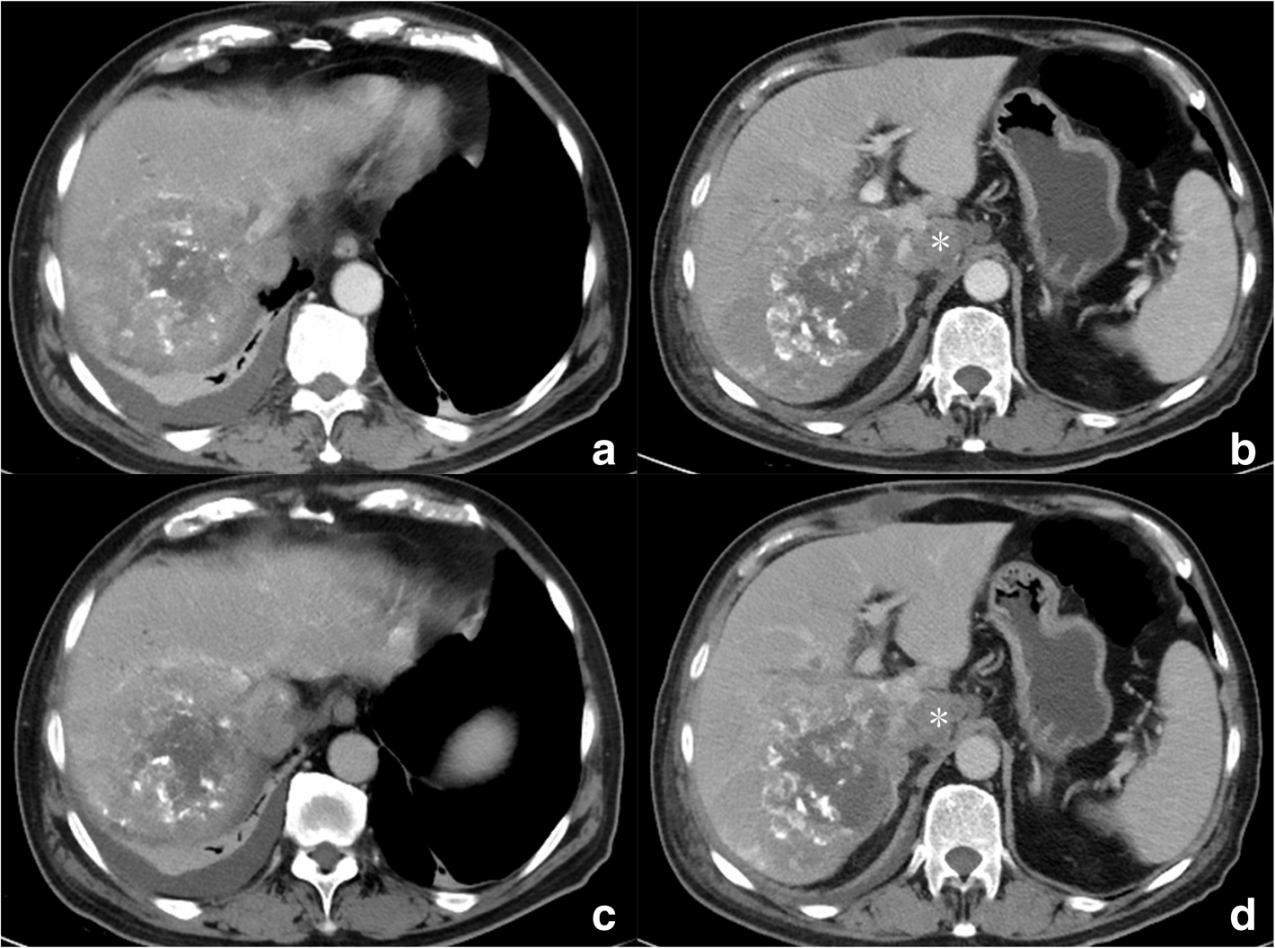
Contrast enhanced CT images of patient 6 who underwent TACE before surgery. **a**, **b** reveals a heterogeneous hypointense mass in right lobe with unclear rim, hyperdense iodine deposition secondary to TACE, and mild enhancement are identified in peripheral portion; **c**, **d** The enhanced portion of the tumor in AP demonstrated washout in PP, the border between tumor and the right adrenal gland is unclear, indicating involvement of right adrenal gland. Hepatic hilar lymphadenopathy is also presented (**b**, **d**.*)

**Fig. 4:**
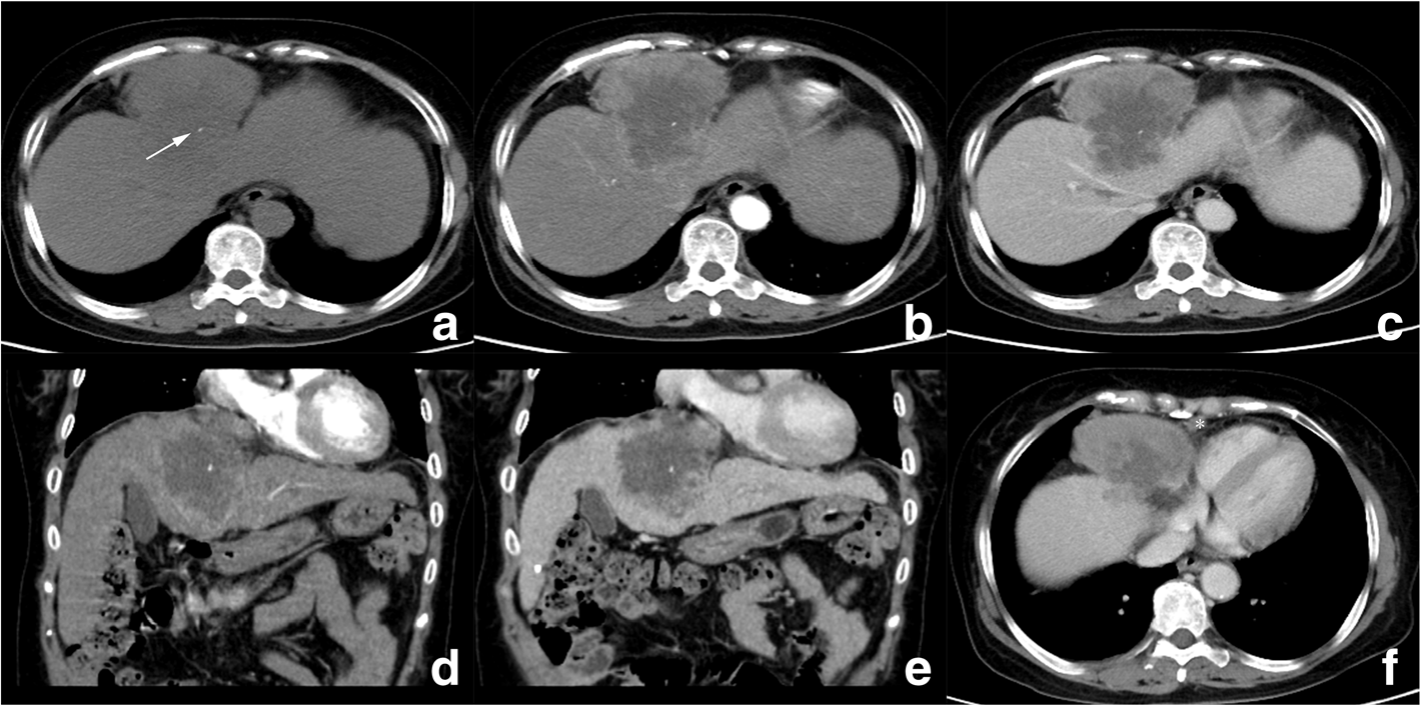
Computed tomography (CT) images of patient 2. **a** Unenhanced CT images reveal a hypodense mass in segment 4 with unclear margin and spot calcification (arrow). Tumor showed significant enhancement in peripheral portion in AP (**b**) and washout in PP (**c**); **d** and **e** are coronal reconstructed PP images, present large central part of cystic degeneration in the tumor; right phrenic angle lymphadenopathy is also observed (**f**, *)

**Fig. 5:**
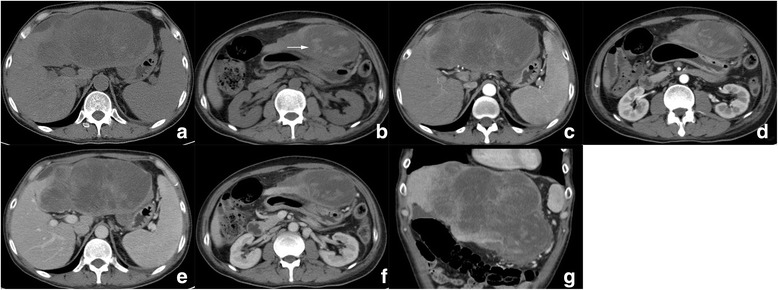
Computed tomography (CT) images of patient 1. **a** Unenhanced CT image shows a large hypodense mass in left lobe; the below image slice shows hyperdense areas in the inferior part of tumor, which represents hemorrhage (**b**, arrow). This tumor presents satellite rim enhancement and small feeding vessel in AP (**c**, **d**) and washout in PP (**e**, **f**). Coronal reconstructed PP image (**g**) shows extensive cystic degeneration

**Fig. 6:**
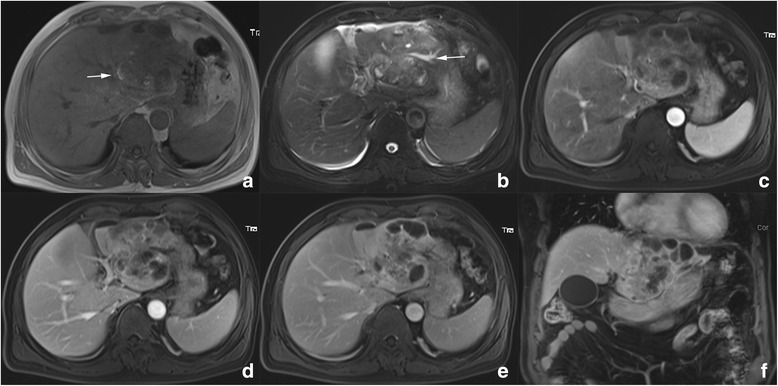
Magnetic resonance (MR) imaging of patient 4. **a** Pre-contrast unenhanced T1WI reveals a heterogeneous hypointense mass in left lobe with spot hemorrhage (arrow). **b** Fat suppressed T2WI shows a heterogeneous hyperintense mass with extensive cystic area, necrosis and hemorrhage. Bile duct dilation and small amount of bilateral pleural effusion is also presented (arrow). **c** Contrast enhanced MR reveal heterogeneous significant enhancement in AP with multiple septations. The enhanced solid portion in AP phase showed washout in PP (**d**) and delay phase (**e**). Coronal reconstructed image form from delay phase well delineates multiple septations in the tumor (**f**)

**Fig. 7:**
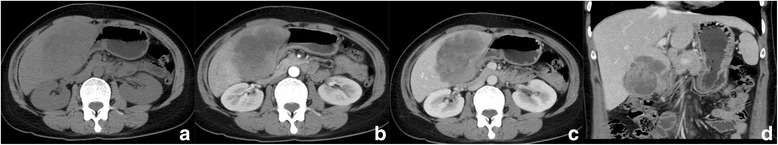
Computed tomography (CT) images of patient 3. **a** Unenhanced CT image shows a hypodense mass in right lobe. The tumor presents moderate enhancement in AP (**b**) and continuous enhancement in PP (**c**) on enhanced images. Coronal reconstructed PP image well delineates heterogeneous texture of the tumor (**d**)

**Fig. 8:**
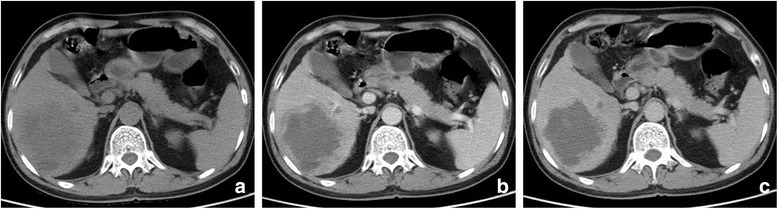
Computed tomography (CT) images of patient 5. **a** Unenhanced CT image reveal a hypodense multilobular mass in right lobe without a clear margin. Contrast enhanced CT reveals rim enhancement in AP (**b**) and washout in PP (**c**)

While five patients have undergone CT evaluation, one (patient 4) had undergone MRI. No.6 patient underwent TACE (Transcatheter arterial chemoembolization) before surgical resection, and only had post-TACE therapy CT images (Fig. 3). The imaging features of six cases of HCS were summarized in Table 3. Four of six (66.7%) tumours were located in the right lobe. All tumours were large bulky masses with extensive central cystic degeneration. Capsule and bile-duct thrombi were not identified in all six tumors. On contrasted enhanced images, tumor presented heterogeneous enhancement, four of six (66.7%) demonstrated significant rim enhancement in AP and washout in PP (Figs. 4, 5, 6 and 7), while the other two tumors (nos. 5, 6) showed moderate rim enhancement in AP and washout in PP (Figs. 3, 8). There were some specific findings in certain tumors, including spot calcification in patient 2 (Fig. 4), hemorrhages in patients 1 (Fig. 5) and 4 (Fig. 6), patchy and satellite hyperdense areas representing iodine deposition post TACE therapy in patient 6 (Fig. 3).

**Table 3:** Preoperative imaging features of 6 cases with hepatic carcinosarcoma

Case NO.	Imaging protocol	Site (segment)	Shape	Margin	Density/intensity	Calcification	Hemorrhage	Cystic/necrotic change	Capsule	Heterogeneity	Enhancement	Imaging diagnosis
1	CT	2,3,4	Multilobular	Clear	Hypo	–	+	+	–	Heterogeneous,	Hyper	HCC
2	CT	4	Multilobular	Unclear	Hypo	+	–	+	–	Heterogeneous	Hyper	HCC
3	CT	5	Bulky	Unclear	Hypo	–	–	+	–	Heterogeneous	Hyper	HCC
4	MRI	2,3	Multilobular	Clear	Hypo-T1WI Hyper-T2WI	–	+	+	–	Heterogeneous	Hyper	CCC
5	CT	5,6	Multilobular	Unclear	Hypo	–	–	+	–	Heterogeneous	Hypo	HCC
6	CT	6,7,8	Bulky	Unclear	Hypo	–	+	+	–	Heterogeneous	Hypo	HCC
Case NO.		Vascular invasion		Nodule enlargement		Invasion into adjacent organs		Bile duct dilation		Bile duct thrombi		Fluids
1		–		–		–		–		–		–
2		–		+		–		+		–		–
3		–		+		+/ Gastric wall and gall bladder		–		–		–
4		–		–		–		+		–		+
5		–		–		–		–		–		–
6		+		+		+/ Right adrenal gland		–		–		+

*Abbreviation*: “+” yes/present/positive, “-” no/absent/negative, *AP* arterial phase, *PP* portal venous phase, *HCC* hepatocellular carcinoma, *CCC* cholangiocellular carcinoma, *Hypo* hypodense/hypointense/hypovascular, *Hyper* hyperdense/hyperintense/hypervascular

Lymphadenopathy were observed in patient 2 (right phrenic angle), 3(hepatic hilar), and 6 (hepatic hilar and para-aortic); peripheral bile-duct mild dilation in patients 2 and 4; satellite metastatic node and right hepatic vein thrombi in patient 6; invasion into the adjacent organs in patient 2 (gastric wall and gall bladder) and 6 (right adrenal gland); pleural effusion in patient 4 and 6.

Unfortunately, because of the lack of awareness of imaging features and the very low morbidity and heterogeneous nature of HCS, all the six tumors were misdiagnosed preoperatively.

## Discussion

In 1989, Craig et al. [19] firstly defined liver carcinosarcomas as hepatic tumors with both an HCC and a non-spindle-cell sarcoma with specialized differentiation, such as malignant cartilage, bone, or skeletal muscle. The WHO defines HCS as a malignant tumor containing an intimate mixture of carcinomatous, either HCC or CCC, and sarcomatous elements [1]. The WHO definition is widely approved, and it is broader than that of Craig et al., because it does not emphasize the specialized differentiation of sarcomatous components. Nevertheless, HCS still remains a rare liver malignancy worldwide. Only 29 cases of HCS have been reported to date [2–18], including the present cases.

Hepatic carcinosarcoma typically arises in adults within the age range of 38 to 76 years, with an average age of occurrence of 55.5 years. The 29 cases of HCS reported to date include 22 men and 7 women, which give a sex ratio of 3:1. The tumor is always large, with a mean diameter of 12.5 cm (range, 3.2 to 25 cm); 23(79.3%) of the 29 patients exhibited tumor diameters> 5 cm. In most cases, symptoms related to the tumor are nonspecific, with right upper abdominal pain and distension reported to be the most common complaints. Of the 29 patients, 15(52.7%) were positive for hepatitis-B surface antigen; 3(31.0%) for hepatitis-C surface antigen; 1 (3.4%, a Western male patient) for hepatitis-D surface antigen; and 9 (13.8%; all Asians) for cirrhosis. While 12 (41.4%) of 29 exhibited elevated serum AFP concentrations, 8 (27.6%), 3 (10.3%), and 2 (6.9%) patients exhibited elevated serum CA19–9, CA125, and CEA concentrations, respectively. Nearly 70% of the patients had advanced stage of HCS at the time of admission. Vascular invasion and thrombi, satellite nodules, and extrahepatic lesions were common (vascular invasion, 7[24.1%]; satellite lesions, 6[20.7%]; and extrahepatic lesions, 11[37.9%]). The most frequent sites of metastasis were the lungs and lymph nodes (hepatic hilar and para-aortic lymph nodes), peritoneum, gall bladder, greater omentum, stomach, diaphragm, and adrenal glands [2–19]. Recurrences were observed in14 (58.3%) of 24 patients with available follow-up data; 12 (50%) patients experienced distant metastasis. A total of 17 (70.8%) patients experienced recurrence and/or metastasis, of whom 14 died within 2 years after surgery. The mean survival time was 11.2 months (range, 1.5–30 months); in 12 (50%) cases, the survival time was less than 11.2 months. All these clinical manifestations suggest that liver carcinosarcoma is highly invasive and metastatic and has poorer prognosis than pure HCC and CCC.

The tumor is usually sharply demarcated and solitary. Histologically, the tumor contains a combination of carcinomatous and sarcomatous elements. The carcinomatous components may include HCC (17/29; 58.6%), CCC (5/29; 17.2%), a combination of both (1/29; 3.4%), or a “not otherwisespecified” adenocarcinoma (7/29; 24.1%), with HCC being the most common. Sarcomatous elements show various levels of differentiation, exhibiting features of undifferentiated spindle cells(10/29; 34.5%), chondrosarcoma (1/29; 3.4%), osteosarcoma (3/29; 10.3%), a combination of chondrosarcoma and osteosarcoma (2/29; 6.9%), leiomyosarcoma (2/29; 6.9%), rhabdomyosarcoma (3/29; 10.3%), fibrosarcoma(6/29; 20.7%), and malignant fibrous histiocytoma (2/29; 6.9%). A specific diagnosis is made on the basis of IHC findings obtained using various antibodies.

The pathogenesis of this rare tumor is controversial. Increasing evidence in recent years [2–15] supports the theory that carcinosarcoma is monoclonal in origin. Most HCS in the present and previous reports had developed in normal livers without cirrhosis. These findings support the theory that the tumor develops from a multipotent hepatic progenitor or stem cell, which then differentiates into both carcinomatous and sarcomatous neoplasms.

Because of its low morbidity, much is unknown about the imaging features of HCS; consequently, accurate preoperative imaging diagnosis is challenging for radiologists. In the present study, all six patients were diagnosed with HCC preoperatively. Given its relatively poor prognosis and highly aggressive bio-behavior, it is crucial to summarize the imaging features of HCS in order to narrow down the differential diagnoses and improve the diagnostic ability. From the present and previous findings, HCS appears to be always very large, occupying more than one segment and even a whole lobe. In the previous reports, the tumors were often bulky, sometimes dumbbell or irregular-shaped, and poorly demarcated in the presence of extrahepatic or organ involvement [2, 4, 6–9, 13, 16–18]. Computed tomography is the most commonly used imaging method for HCS. All the reported tumors exhibited hypodensity on plain CT images. Tumor heterogeneity is secondary to the complicated components and extensive necrosis. In the present study, cystic changes and necrotic areas were located in the central region, with solid portions in the periphery. Among the previous 10 cases of HCS with available imaging features (Table 4) and the present 6 cases (Table 3), calcification and osteogenesis were observed in 5(31.3%) cases. Osteogenesis is a specific differentiation feature for HCS with osteosarcomatous elements, given that HCC and CCC never exhibit osteogenesis. The previous studies [2, 3, 6, 14, 16, 18] revealed that a large heterogeneous tumor with osteogenesis is highly suggestive of HCS. However, osteogenesis is not frequently observed in HCS, because it only presents in HCS included osteosarcoma or chondrosarcoma. Unfortunately, our six cases lack of osteosarcoma or chondrosarcoma component, osteogenesis is not presented, which make the imaging not so specific. Intratumoral hemorrhage is common and observed in 8 (50.0%) of 16 cases; this feature exhibits hyperdensity on plain CT images, hyperintensity on T1W images, and hypointensity on T2W images. In contrast to HCC, HCS rarely exhibits a capsule; only 6 tumors with diameters< 5.0 cm (6/29; 20.7%) have been reported as exhibiting capsules [13, 19]. The absence of a capsule in most HCS might be because of their highly aggressive behavior.

**Table 4:** Imaging features of 10 previously reported cases of hepatic carcinosarcoma

Reference NO.	Case NO.	Protocol	Density/intensity	Cystic/necrotic change	Calcification	Hemorrhage	Margin	Shape	Capsule	Vascular invasion	Bile duct dilation/ thrombi	Organ involvement	Nodule metastasis	Ascites	Enhancement appearance
4	1	CT	Hypo	+	+	–	Clear	Multiloculated	–	–	–	–	–	+	NA
6	2	CT, MRI	Hypo Hypo-TWI, hyper-T2WI	+ +	+ -	+	Unclear	Multiloculated	–	–	–	–	–	–	Hyper
7	3	CT	Hypo	+	NA	+	Unclear	Rugby ball	–	–	–	+	+	+	Hyper
8	4	MRI	Hypo-T1WI Hyper-T2WI	+	–	+	Clear	Multiloculated	–	–	–	–	+	–	Hyper
9	5	CT	Hypo	+	–	–	Clear	Bulky	–	–	–	–	+	–	Hyper
13	6	CT	Hypo	+	+	–	Clear	Round	+	–	–	–	+	–	Hyper
14	7	CT, MRI	Hypo Hypo-T1WI, Hyper-T2WI	+	+	+	Unclear	Bulky	–	–	Dilation	+	NA	–	Hyper
16	8	CT,MRI	Hypo-TWI, Hyper-T2WI	+	+	+	Unclear	Bulky	–	–	–	+	NA	NA	NA
17	9	CT	Hypo	+	–	–	Unclear	Bulky	–	–	Thrombi	–	–	–	Hyper
18	10	CT	Hypo	+	–	–	Clear	Bulky	+	–	–	–	–	–	Hyper

*Abbreviation*: “+” yes/present/positive, “-” no/absent/negative, *NA* not available, *Hypo* hypodense/hypointens/hypovasculare, *Hyper* hyperdense/hyperintense/hypervascular

Most HCS are very large and present extra-hepatic lesions at diagnosis. The enhancement pattern is determined by the intratumoral components. All tumors in the previous studies [4, 6–9, 13, 14, 16–18] exhibited heterogeneous and hypervascular enhancement, i.e., rim enhancement in AP and washout in delay phase. However, in patients 5 and 6 in the present study, the tumors exhibited mild to moderate rim enhancement on CT images; in case 6, this hypovascularity on enhanced images may due to TACE therapy which decreased tumor angiogenesis. In case 5, pathologically, the sarcomatous element in the central part was very large and small amount of epithelial element (HCC) exhibited satellite distribution at the tumor rim, the ratio of tumor component determines its moderate rim enhancement. Besides, in these two cases, the sarcomatous element exhibited undifferentiated spindle cells and differentiated leiomyosarcoma, respectively; the histopathologic results revealed few vascular components and IHE results revealed negative CD34 expression in the two tumors.

At present, diagnosis of HCS largely depends on pathological findings. Tomographic diagnosis of HCS is not often attempted because of the difficulty in arriving at a correct differential diagnosis of other liver malignancies. Differential diagnoses often include HCC, CCC, and sarcoma. Hepatocellular carcinoma is the most common primary malignant tumor of the liver, predominantly occurring in the setting of post-hepatitis cirrhosis [20]; it is commonly observed in senior male patients. It usually presents as a hypodense mass of varying diameters and hypervascularity on enhanced images. Satellite nodules and vessel thrombi are common in HCC. These tumors, especially well-differentiated ones, always exhibit a capsule at the rim. Mass-forming CCC originates from bile-duct epithelium and is usually located in the left lobe. Most CCC involves senior male subjects with or without cirrhosis. Typical imaging features include homogeneous attenuation/intensity, irregular peripheral enhancement with gradual centripetal enhancement, capsular retraction due to abundant central fibrosis, and peripheral bile-duct dilation [21]. In contrast to HCS, both HCC and CCC rarely exhibit cystic changes, necrosis, or calcification; these features, if present, are always punctuated or spotted in appearance. Yasutake et al. [14] reported a case of liver carcinosarcoma including an HCC-component, which was hyperintense in the hepatobiliary-phase on gadolinium-ethoxybenzyl-diethylenetriamine pentaacetic acid (Gd-EOB-DTPA) contrasted enhanced MRI, Thus, they concluded that hyperintensity of a lesion in the hepatobiliary-phase adds in the differential diagnosis. However, the role of Gd-EOB-DTPA enhanced MRI in distinguishing HCS from HCC needs more studies to validate, because no other similar reports and experiences have been reported. Primary liver sarcoma is very rare, representing < 1% of liver malignancies; it predominantly occurs in childhood. Metastatic sarcomas are more common in adults. A major characteristic of metastatic sarcomas is that they are often very large heterogeneous tumors without clear borders on CT and MR images [10, 22]. The intratumoral component of these tumors is relatively complicated, and a cystic or cystic–solid, mucinous appearance is common. Enhancement characteristics depend on histodifferentiation; most tumors present delayed, mild to moderate, heterogeneous enhancement [23, 24]. Metastasis and extrahepatic invasion are relatively common. Imaging features of HCS partly overlap with those of HCC, CCC, and sarcoma. Extensive calcification and osteogenesis is specific for diagnosis of HCS with osteosarcomatoid differentiation. Hypervascularity in the AP might indicate HCS rather than sarcoma. However, definitive diagnosis of HCS is based on the combination of clinical, radiological, and histopathological evidences.

Surgical resection appears to be the most effective treatment for HCS. Although radical resection is still recommended [2], the presence of extrahepatic metastases, vessel involvement, and/or invasion of surrounding organs renders curative resection impossible. Careful follow-up and postoperative intervention, including radiotherapy and chemotherapy, might be beneficial for extending disease-free survival [4, 7, 11, 18].

## Conclusions

The clinical and imaging features of HCS lack specificity except osteogenesis in certain histological pattern. Although specific osteogenesis was not shown in this series of six cases, the detailed clinical and imaging features may be helpful to improve the familiarity of this rare tumor. A large tumor with more extensive cystic and necrotic degeneration, without capsue, with hypervascular enhancement, more frequently presented with lymphadenopathy and invasion of adjacent organs, these findings may be helpful to distinguish HCS from other malignancy. Due to the heterogeneous nature and very low morbidity of HCS, a more accurate preoperative diagnosis depends on the combination of clinical features and careful imaging observation. Given its aggressive nature and poor prognosis, HCS requires treatment by radical surgical resection and careful follow-up with CT or MRI.
